# Global law, policy, and governance for effective prevention and control of COVID-19: A comparative analysis of the law and policy of Pakistan, China, and Russia

**DOI:** 10.3389/fpubh.2022.1035536

**Published:** 2023-01-04

**Authors:** Muhammad Bilawal Khaskheli, Shumin Wang, Rana Yassir Hussain, M. Jahanzeb Butt, XiaoShan Yan, Sara Majid

**Affiliations:** ^1^School of Law, Dalian Maritime University, Dalian, Liaoning, China; ^2^Division of Management and Administrative Science, UE Business School, University of Education, Lahore, Pakistan; ^3^School of Maritime Management, Dalian Maritime University, Dalian, Liaoning, China; ^4^School of Philosophy, Zhejiang University, Hangzhou, China; ^5^School of Management, Xi'an Jiaotong University, Xi'an, Shaanxi, China

**Keywords:** global health policy, global health governance, global health law, pandemic law, COVID-19 law, Pakistan epidemic prevention law, China epidemic prevention law, Russia epidemic prevention law

## Abstract

Global health governance is a developing system in this complex institutional regime. The local and regional health policies sometimes challenge global health governance due to diverse discourse in various countries. In the wake of COVID-19, global health governance was reaffirmed as indifferent modules to control and eliminate the pandemic; however, the global agencies later dissected their own opinion and said that “countries must learn to live with a pandemic.” Given the controversial statement, this research focuses on the strong and effective policies of the Russian Federation, Pakistan, and China. The research uses the law and governance results and newly developed policies of the three countries formed under the global health policies. The conclusion is based on the statement that in order to live with the pandemic, strong health measures are required at each level.

## 1. Introduction

On December 27, 2019, a cluster of unexplained pneumonia cases was traced to the Huanan Seafood Market in Wuhan, Hubei's Provincial capital (1). The epidemic, at that time, commonly referred to as coronavirus (later termed COVID-19), was spreading globally in the form of a pandemic (2). Scientifically, this disease was one form of the commonly found coronaviruses in birds' genome sequencing, also known as severe acute respiratory syndrome coronavirus 2 (SARS-CoV-2). The general public was unaware of specific coronaviruses at the time and could not take prompt action to stop that from spreading because the virus was disseminated by droplets and direct touch (3). As it began in China, China was among the first countries to explore the disease and its viral infection, as well as one of the first to take drastic measures in response to the outbreak, including “lockdowns,” required to wear face mask restrictions, quarantine, and working at home to meet the new challenge of the COVID-19 pandemic during in the spread of the virus and to bring the emergence and spread under control (4).

On January 30, 2020, the World Health Organization (WHO) designated COVID-19 as a global health case of emergencies of international significance (otherwise known as a global health emergency) (5). Across the borders, COVID-19 was spreading rapidly; thus, on March 11, the same year, the WHO situation report−79 declared COVID-19 as a pandemic (6). In the following month, the outbreak claimed some 79,235 lives globally, which appears to be nearing an end in China, where it was first reported (7). However, it keeps spreading in Europe, the United States, and other regions of the world, including several low- and middle-income countries (LMICs) (8). Furthermore, it is easily spread in densely populated areas, particularly entertainment venues and public facilities (such as cruise ships, ships, and airplanes) (9). The epidemic has prompted extraordinary global responses, including travel restrictions, incarceration, and lockdown measures that have all been implemented in several countries (10). These actions were implemented in an “urgent situation” manner and are mostly reactive, with the goal of limiting disease transmission while waiting for a particular treatment and/or vaccine to be produced during an outbreak (11). According to WHO, COVID-19 has been associated with more than 179 million infections and 3.89 million confirmed fatalities, from December 2019 to March 2022 (12).

Although the responses and policy measures taken by WHO remained and are still applaudable at a global level, the control measures taken within individual countries are highly criticized (13). The best practices adopted through emergency law and policy in response to COVID-19 were Pakistan, China, and Russia (14–16). These countries' practices through law and policy were acclaimed by WHO, regional health agencies and influential countries (17). The basic aim of this research focuses on the main question related to the kind of emergency law and policy response to COVID-19 adopted by these three countries. Moreover, how do global health policy and governance work during an outbreak, local and international contained COVID-19? COVID-19 is the most severe outbreak in our lifetimes and reminds us why the infectious disease has been central to global health governance in the past and will remain so for the foreseeable future. COVID-19 is a novel ailment that not only poses problems for medical experts treating it but also forces international leaders and politicians to find new measures to combat its far-reaching implications (18). Because a pandemic of this magnitude poses unprecedented issues in terms of maintaining order, medical facility availability, global health governance, and social and financial security for all, new legislation is being developed to guide us through this unknown region (19). Some countries have passed new laws to deal with the COVID-19 epidemic and its devastating effects (19).

COVID-19 emergency legislation during COVID-19 worldwide. Recently, the variants of COVID-19 became extremely contagious, wherefore, Pakistan's continued spread of the extremely dangerous Omicron coronavirus strain was not taken seriously (20). On the other hand, South Africa and the United Kingdom are among those countries that have declared their intentions to relax the bulk of COVID-19 prohibitions (21). Therefore, some regions' global health officials and sustainability leaders have argued that the pandemic is entering a new phase and urged populations to begin “learning to live with COVID-19” (22). The flurry of announcements, which include plans to scrap COVID-19 self-isolation for those who test positive in England and end social distancing rules in the Netherlands, come as the pandemic enters a new phase. Pakistan has eliminated all coronavirus-related restrictions across the nation, claiming that the outbreak has ended. The National Command and Operation Center (NCOC) for COVID-19 in Pakistan decided that all limitations linked to the coronavirus would be applied (23). The goal of this study was to determine the components involved in COVID-19 in various situations to assess the impact of interventions on COVID-19 spread. This study also reveals some of the most effective strategies for countries to combat pandemic, control new forms of infectious illness, change at the WHO, a comparison of global goal governance and health policy and other new legislation that emerges during outbreaks (24, 25).

## 2. Materials and methods

### 2.1. Data sources

The data collected for this research includes data from the official websites of the countries and WHO and our World data, PubMed and web of science. The literature applied in this study looked at research published in English in peer-reviewed journals on the databases mentioned above from January 2020 to August 2022, following keywords [global health policy, global health governance, global health law, pandemic law, COVID-19 law, Pakistan epidemic prevention law, China epidemic prevention law, Russia epidemic prevention law, and World Health Organization (WHO)] and daily case studies. The major focus was on comparison studies with some major countries that effectively adopted new laws, regulations, and global health policies to be analyzed among them. The International Health Regulations (IHR) include Article 25 of the Universal Declaration on Human Rights (UDHR), Article 28 and 64 of WHO Conventions, the 1374 Venice Convention for Quarantine for Plague (VCQP), the 1851 Paris Convention de First International Sanitary Conference, the 1947 Geneva WHO Epidemiological Information Service (WHO-EIS), the 1951 Geneva International Sanitary Regulations, and the 1969 Geneva International Health Regulations (GIHR) (26–30). The given regulations, conventions and treaties are placed under the scope and transparency of the WHO's pandemic declarations of a public health emergency of international concern (PHEIC) during the COVID-19 outbreaks worldwide (31).

### 2.2. Literature review

To fill in the literature's gaps, a systematic sustained ethical analysis was deemed appropriate to synthesize and integrate relevant literature with a lack of comprehensive understanding of the research questions. The review sought to identify the breadth, weight, and gaps in the body of existing literature review in order to inform future study and practice. The following keywords that were used were global law, policy governance, and control of COVID-19 between Pakistan, China, and Russia. We included any study that addressed the ethics imperative principle in guiding resource allocation to first responders or health care workers, global health governance, COVID-19 law, and epidemic prevention laws in those countries. The review looked at the ethical concerns and problems found in the literature to find the ethical practices that were important for the COVID-19 response and a previous search of academic literature yielded the following results, which are listed below.

#### 2.2.1. Global health policy

Given above, the basic international law for COVID-19 is Article 25 of UDHR, effectively stating that medical care is the right of each global citizen (32). Further, the VCQP, WHO-EIS, and GIHR endorse the effective implementation of global health governance during the pandemic. The COVID-19 pandemic has shaken many nations' faith in the efficacy of existing preparation concepts and institutions to appropriately alleviate global health catastrophes (33). Recent research has revealed considerable discrepancies in how governments comprehended and responded to the epidemic to limit its implications, leading to huge country variances in fatality rates and economic ramifications (33). Furthermore, since the late 1990's, the WHO has been attempting to strengthen and standardize national pandemic preparation (34). Ahead evaluations of pandemic preparation' insufficiency in anticipating COVID-19 effectiveness have previously been shown, such as the 2019 Global Health Security Index (GHSI), which put the US and the UK first and second in the world in terms of health security capabilities before the pandemic (35). Due to the US and UK's first COVID-19 answers, forecasts of rankings such as the GHSI have received substantial criticism (36). A good way to investigate these challenges is to examine how pandemic preparation mechanisms established and coordinated by the WHO-IHR were employed in countries with similar public health systems (37).

Vaccination Nationalism By the end of March 2021, the WHO had recorded more than 120 million cases and more than two million fatalities globally (38). According to the WHO Director-General, 39 million doses of the COVID-19 vaccination had been provided in 49 industrialized nations as of January 18, 2021, with just 25 doses administered in developing countries (39). There are just 25 dosages, not 25,000,000 or 25,000. In the meantime, the situation has altered, but large disparities exist between industrialized and developing nations (40). Some governments, including the United States of America, the United Kingdom, and the European Union, have sought to buy (monopolize) the entire production of candidate vaccines, or to prevent their export outside their borders, to cover their populations first and foremost, a practice known as “vaccine nationalism” (41). For example, the United States has signed at least six bilateral agreements totaling more than one billion doses, more than enough to immunize its whole population (328 million) (41). According to Duke University's Center for Global Health Innovation, the European Union (447 million), the United Kingdom (67 million), and Canada (37 million) have inked seven bilateral agreements, with the potential to cover their populations twice, four times, and six times over (42). Vaccine shortages, caused by production challenges, have resulted not just in a competitive market with uneven distribution but also in geopolitical leverage games, known as “vaccine diplomacy” (43). The Chinese vaccination Sinovac, for example, has reached Brazil, the Russian vaccine Sputnik has reached Argentina, and the Indian vaccine Covishield (developed in collaboration with Oxford-AstraZeneca) has reached various nations in the Global South (44). As shown in Table 1, the COVID-19 Health System Response Monitor (HSRM) is headed by the technical competence of the following countries' contributors (45, 46).

**Table 1:** World Health Organizations location by the COVID-19.

Albania	Gazmend Bejtja, WHO Regional Office for Europe, Country Office Bettina Menne, WHO Regional Office for Europe, Country Office Adrian Xinxo, WHO Regional Office for Europe, Country Office	Canada	Sara Allin, North American Observatory on Health Systems and Policies; University of Toronto Tiffany Fitzpatrick, University of Toronto Michel Grignon, McMaster University Nessika Karsenti, Schulich School of Medicine, Western University Madeline King, North American Observatory on Health Systems and Policies; Telfer School of Management, University of Ottawa Anna Kurdina, University of Toronto Greg Marchildon, North American Observatory on Health Systems and Policies; University of Toronto Monika Roerig, North American Observatory on Health Systems and Policies; University of Toronto Sterling Stutz, University of Toronto
Armenia	WHO Regional Office for Europe, Country Office	Denmark	Allan Krasnik, University of Copenhagen Hans Okkels Birk, University of Copenhagen Signe Smith Jervelund, University of Copenhagen Karsten Vrangbaek, University of Copenhagen
Austria	Florian Bachner, National Public Health Institute Katharina Habimana, National Public Health Institute Anita Haindl, National Public Health Institute Sonja Neubauer, National Public Health Institute Andrea Schmidt, National Public Health Institute	Croatia	Maja Banadinovic, School of Public Health Andrija Štampar, University of Zagreb Aleksandar Dzakula, School of Public Health Andrija Štampar, University of Zagreb Iva Miloš, School of Public Health Andrija Štampar, University of Zagreb Maja Vajagic', School of Public Health Andrija Štampar, University of Zagreb Sterling Stutz, University of Toronto
Azerbaijan	WHO Health Emergencies Programme	Estonia	Triin Habicht, WHO Barcelona Office for Health Systems Strengthening Kristiina Kahur, Private consultant Kaija Kasekamp, Ministry of Social Affairs Kristina Köhler, WHO Regional Office for Europe Marge Reinap, WHO Regional Office for Europe, Copenhagen Andres Vork, University of Tartu, Johan Skytte Institute of Political Studies
Belarus	Batyr Berdyklychev, WHO Regional Office for Europe, Country Office Andrei Famenka, WHO Regional Office for Europe, Country Office Viatcheslav Grankov, WHO Regional Office for Europe, Country Office	Belgium	Sophie Gerkens, Belgian Health Care Knowledge Center Karin Rondia, Belgian Health Care Knowledge Center
France	Coralie Gandré, The Institute for Research and Information in Health Economics (IRDES) Zeynep Or, The Institute for Research and Information in Health Economics (IRDES)	Finland	Salla Atkins, University of Tampere Vesa Jormanainen, Finnish Institute for Health and Welfare (THL) Ilmo Keskimäki, Finnish Institute for Health and Welfare (THL) Meri Koivusalo, University of Tampere Pauli Rautiainen, Finnish Institute for Health and Welfare (THL) Eeva Reissell, Finnish Institute for Health and Welfare (THL) Markku Satokangas, Finnish Institute for Health and Welfare (THL) Liina-Kaisa Tynkkynen, University of Tampere Marjaana Viita-aho, University of Tampere
Czech Republic	Lucie Bryndová, Institute of Economic Studies, Charles University Adam Poloček, Charles University, Czech Republic Tomáš Roubal, WHO Regional Office for Europe, Country Office Andrea Silenzi, Università Cattolica del Sacro Cuore Walter Ricciardi, Fondazione Policlinico Universitario A. Gemelli	Georgia	Silviu Domente, WHO Regional Office for Europe, Country Office Tamila Zardiashvili, WHO Regional Office for Europe, Country Office
Kazakhstan	Dana Abeldinova, WHO Regional Office for Europe, Country Office Serzhan Aidossov, WHO Regional Office for Europe, Country Office Nadira Yessimova, WHO Regional Office for Europe, Country Office	Montenegro	Senad Begić, WHO Regional Office for Europe, Country Office Mina Brajović, WHO Regional Office for Europe, Country Office Nemanja Radojević, WHO Regional Office for Europe, Country Office Batrić Vukč ević, WHO Regional Office for Europe, Country Office
Greece	Charalampos Economou, Panteion University of Social and Political Sciences Daphne Kaitelidou, National and Kapodistrian University of Athens Olympia Konstantakopoulos, National and Kapodistrian University of Athens Lilian Venetia Vildiridi, Ministry of Health	Kyrgyzstan	Aliina Altymysheva, WHO Regional Office for Europe, Country Office Nazira Artykova, WHO Regional Office for Europe, Country Office Tasnim Atatrah, WHO Regional Office for Europe, Country Office Akbar Esengulov, WHO Regional Office for Europe, Country Office Kaliya Kasymbekova, WHO Regional Office for Europe, Country Office Monolbaev Kuban, WHO Regional Office for Europe, Country Office Moldoisaeva Saltanat, WHO Regional Office for Europe, Country Office Salieva Saltanat, WHO Regional Office for Europe, Country Office Aigul Sydykova, WHO Regional Office for Europe, Country Office Nurshaim Tilenbaeva, WHO Regional Office for Europe, Country Office
Hungary	Peter Gaal, Semmelweis University, Health Services Management Training Center Viktoria Szerencses, Semmelweis University, Health Services Management Training Center Zita Velkey, Semmelweis University, Health Services Management Training Center	Latvia	Daiga Behmane, Riga Stradins University Janis Misinš, Riga Stradins University
Iceland	Sigurbjörg, Sigurgeirsdóttir, University of Iceland	Lithuania	Laura Miščikiene, Lithuanian University of Health Sciences Agne Slapšinskaite, Lithuanian University of Health Sciences Mindaugas Štelemekas, Lithuanian University of Health Sciences
Ireland	Sarah Barry, The Center for Health Policy and Management, School of Medicine, Trinity College Dublin Sara Burke, The Center for Health Policy and Management, School of Medicine, Trinity College Dublin Rikke Siersbaek, The Center for Health Policy and Management, School of Medicine, Trinity College Dublin Malgorzata Stach, The Center for Health Policy and Management, School of Medicine, Trinity College Dublin Steve Thomas, The Center for Health Policy and Management, School of Medicine, Trinity College Dublin	Luxembourg	Juliane Winkelmann, University of Technology, Berlin/European Observatory on Health Systems and Policies
The Netherlands	Peter Groenewegen, NIVEL—Netherlands Institute for Health Services Research Judith de Jong, NIVEL—Netherlands Institute for Health Services Research Madelon Kroneman, NIVEL—Netherlands Institute for Health Services Research John Paget, NIVEL—Netherlands Institute for Health Services Research	North Macedonia	Simona Atanasova, WHO Regional Office for Europe, Country Office Margarita Spasenovska, WHO Regional Office for Europe, Country Office Jihane Tawilah, WHO Regional Office for Europe, Country Office
Poland	Katarzyna Badora-Musiał, Institute of Public Health, Jagiellonian University Krakow Maciej Furman, Institute of Public Health, Jagiellonian University Krakow Małgorzata Gałazka-Sobotka, Lazarski University Rafał Halik, National Institute of Public Health Iwona Kowalska-Bobko, Institute of Public Health, Jagiellonian University Krakow Magdalena Kozela, National Institute of Public Health Kamila Parzonka, National Institute of Public Health Christoph Sowada, Institute of Public Health, Jagiellonian University Krakow Marzena Tambor, Institute of Public Health, Jagiellonian University Krakow	Norway	Haldor Byrkjeflot, University of Oslo Vegard Skau Ilseth, Norwegian Directorate of Health Anne Karin Lindahl, University of Oslo Ingrid Sperre Saunes, Norwegian Institute of Public Health
Israel	Shuli Brammli-Greenberg, Braun School of public health, the Hebrew University of Jerusalem and Myers-JDC-Brookdale Institute Amit Meshulam, Myers-JDC-Brookdale Institute Gideon Leibner, The Hebrew University of Jerusalem Nadav Penn, Myers-JDC-Brookdale Institute Ruth Waitzberg, Myers-JDC-Brookdale Institute, Ben Gurion University of the Negev, Israel; Technical University of Berlin, Germany	Malta	Malta Public Health COVID-19 Response Team, University of Malta and Ministry of Health
Italy	Giovanni Fattore, Bocconi University Antonio Giulio de Belvis, Università Cattolica del Sacro Cuore Alisha Morsella, Università Cattolica del Sacro Cuore Gabriele Pastorino, WHO Regional Office for Europe Andrea Poscia, Università Cattolica del Sacro Cuore Amélie Schmitt, WHO Regional Office for Europe, Country Office Melita Vujnovic, WHO Regional Office for Europe, Country Office Elena Dmitrievna Yurasova, WHO Regional Office for Europe, Country Office	Republic of Moldova	Oxana Domenti, WHO Regional Office for Europe, Country Office Iuliana Garam, WHO Regional Office for Europe, Country Office Stela Gheorgita, WHO Regional Office for Europe, Country Office Igor Pokanevych, WHO Regional Office for Europe, Country Office
Romania	Silvia Gabriela Scintee, National School of PublicHealth Dana Farcasanu, Center for Health Policy and Services	Russian Federation	Aleksandr Goliusov, WHO Regional Office for Europe, Country Office Tufan Nayir, WHO Regional Office for Europe, Country Office Irshad A. Shaikhi, WHO Regional Office for Europe, Country Office
Monaco	Delphine Lanzara, Ministry of Health Julie Malherbe, Ministry of Health Enrique Bernal-Delgado, Health Services and Policy Research Unit. Institute for Health Sciences in Aragon (IACS) Francisco Estupiñán-Romero, Health Services and Policy Research Unit. Institute for Health Sciences in Aragon (IACS)	Slovakia	Martin Smatana, Private consultant (formerly Ministry of Health)
San Marino	Alessandra Melini, State Authority for Health and Social Security Gabriele Rinaldi, State Authority for Health and Social Security	Slovenia	Tit Albreht, National Institute of Public Health
Serbia	Aleksandar Bojovic, WHO Regional Office for Europe, Country Office Miljan Rancic, WHO Regional Office for Europe, Country Office Ivan Zivanov, WHO Regional Office for Europe, Country Office	Spain	Ester Angulo-Pueyo, Health Services and Policy Research Unit. Institute for Health Sciences in Aragon (IACS)
Sweden	John-Erik Bergkvist, Swedish Agency for Health and Care Services Analysis (Vårdanalys) Kerstin Gunnarsson, Swedish Agency for Health and Care Services Analysis (Vårdanalys) Alexander Hedlund Kancans, Swedish Agency for Health and Care Services Analysis (Vårdanalys) Nils Janlöv, Swedish Agency for Health and Care Services Analysis (Vårdanalys) Simon Jehrlander, Swedish Agency for Health and Care Services Analysis (Vårdanalys)	Ukraine	Jarno Habicht, WHO Regional Office for Europe, Country Office Nataliia Piven, WHO Regional Office for Europe, Country Office
United Kingdom	Natasha Curry, The Nuffield Trust Selina Rajan, London School of Hygiene and Tropical Medicine	USA	Matthew Alexander, Virginia Commonwealth University School of Medicine Andriy Koval, Department of Health Management and Informatics, University of Central Florida Lynn Unruh, Department of Health Management and Informatics, University of Central Florida
Uzbekistan	WHO Health Emergencies Programme	Switzerland	Stefan Boes, University of Lucerne Sarah Mantwill, University of Lucerne Tanya Kasper Wicki, University of Lucerne
Switzerland	Stefan Boes, University of Lucerne Sarah Mantwill, University of Lucerne Tanya Kasper Wicki, University of Lucerne	Turkey	Çetin Dikmen, WHO Regional Office for Europe, Country Office Toker Erguder, WHO Regional Office for Europe, Country Office Berk Geroglu, WHO Regional Office for Europe, Country Office

#### 2.2.2. Global health diplomacy

Health diplomacy is described as “international aid or collaboration aimed to improve health, or that uses health programs to advance non-health-related foreign interests” is how health diplomacy is described (47–49). States, international multilateral organizations like the Group of 20 (G20), and private philanthropic organizations like the Bill and Melinda Gates Foundation can all engage in health diplomacy (50, 51). States frequently collaborate with global intergovernmental organizations such as the World Health Organization (WHO), non-governmental organizations (NGOs), and pharmaceutical corporations to improve their global health policies, blurring this divide (52, 53). The disciplinary borders of health diplomacy are also blurry since they combine public health, international relations, management, law, and economics (54). Finally, health diplomacy is transnational in character since it “relates to health challenges and factors that transcend national boundaries, are worldwide in origin, and require global consensus to solve” (55). “Vaccine diplomacy” is another word that gained popularity during the epidemic (56). It is an all-encompassing term that refers to nearly every area of global health diplomacy based on vaccinations (57). It focuses on international organizations and non-governmental organizations. Vaccine diplomacy may be used to save lives and stop wars, which is a large component of it (57).

Vaccine science diplomacy is a subset of vaccine diplomacy that is a hybrid of components of global health diplomacy and science diplomacy, referring to the cooperative creation of life-saving vaccines and related technology with scientists from ideologically opposed or even hostile governments (58). With the spread of the COVID-19 pandemic, the term “coronavirus diplomacy has begun to be used, particularly in relation to China, as part of the country's broader public relations strategy, which is supposed to counteract negative perceptions of this country while also presenting it as a responsible citizen of global society” (59). Coronavirus diplomacy consists of two phases: a first emergency phase marked by so-called mask diplomacy (medical assistance delivery) and a second phase marked by the diplomatic use of vaccinations, which has evolved into vaccine geopolitics (60). As a result, Russia has grown in importance as a result of the creation of Sputnik V, the world's first vaccine. On the other hand, China looks to be the most active nation in this area (61).

The virus can be as hazardous as a lack of reliable information and trusted sources. Not only can false information mislead people, but it may also put people's lives in peril by inciting them to disregard public health advice, use unapproved treatments, or refuse vaccines, and all of this allows numerous actors to propagate distorted information for geopolitical purposes (62). According to EUvsDisinfo, the Russian effort to market the Sputnik V vaccine evolved into a whole-of-government strategy between December 2020 and the first quarter of 2021, including state institutions, state corporations, and the media in practically daily interventions (62). A focus has accompanied Sputnik V's promotion on the EU's inefficiency, science research delays, and divisions among EU members. The pro-Kremlin media has been very critical of the European Medicines Agency (EMA) for taking too long to review the Sputnik V vaccine and for political bias (63).

During the emergency period, the Kremlin provided COVID-19-related assistance to a number of nations throughout the world, including Italy, Serbia, and Bosnia and Herzegovina (64). Russia faced competition from China's own mask diplomacy in some countries, such as those engaged in the Belt and Road Initiative and the 17+1 platform in Eastern Europe (65). For example, Russia has pledged to deploy 11 military planes with medical equipment to Serbia, the largest Balkan country and a key friend of Moscow (66). China helped as well, sending supplies and equipment. President Vucic praised Vladimir Putin on Twitter, saying the two nations' friendship had been “reaffirmed” (67). The Chinese President, on the other hand, had a far warmer reception, with a big billboard in central Belgrade proclaiming “Thank you, Brother Xi!” and other expressions of Sino-Serbian ties, such as assertions by the previous Chinese ambassador that Serbia and China are really one family (68). During the second phase of the pandemic, Russia was able to fulfill foreign policy aims by using the world's first registered vaccine, Sputnik V. As of November 2021, the vaccine was approved in 70 countries throughout the world. Sputnik V began competing in the worldwide market shortly after it was registered in August 2020. Still, the other two Russian-produced vaccines licensed at the time of writing, EpiVacCorona and CoviVak, are primarily intended for local use (69).

## 3. Results design

### 3.1. Responses to COVID-19

**Figure 1:**
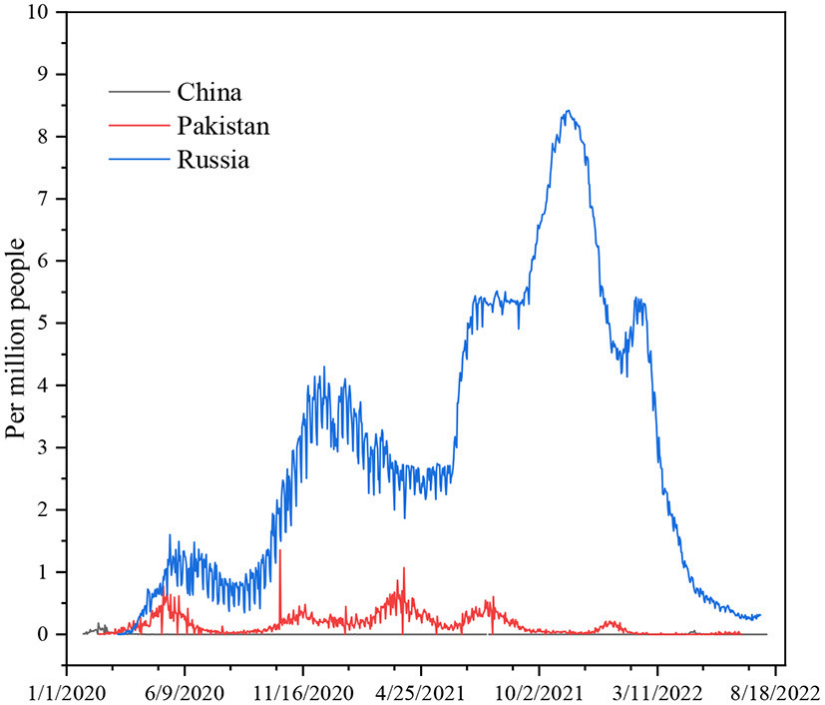
Daily new confirmed COVID-19 deaths per million people. Data source: https://github.com/CSSEGISandData/COVID-19, raw data on confirmed cases and deaths for all countries is sourced from the COVID-19 data repository by the Center for Systems Science and Engineering (CSSE) at Johns Hopkins University; our complete COVID-19 dataset is a collection of the COVID-19 data maintained by Our World in Data. It is updated daily and includes data on confirmed cases, deaths, hospitalizations, and testing. Variable time January 27, 2020–August 20, 2022.

**Figure 2:**
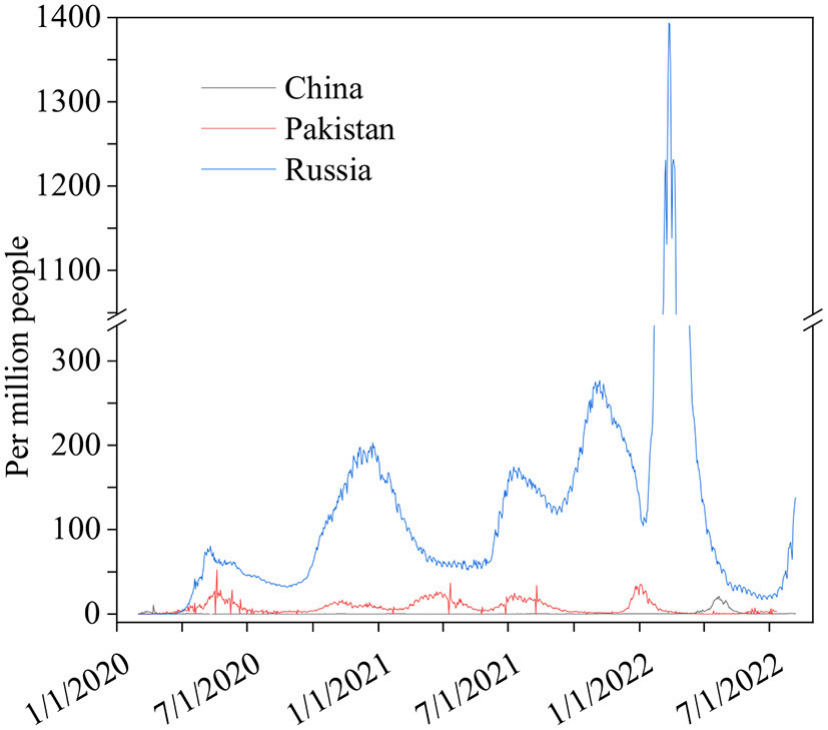
Daily new confirmed COVID-19 cases per million people. Data source: https://github.com/CSSEGISandData/COVID-19, raw data on confirmed cases and deaths for all countries is sourced from the COVID-19 data repository by the Center for Systems Science and Engineering (CSSE) at Johns Hopkins University. January 27, 2020–August 20, 2022.

**Figure 3:**
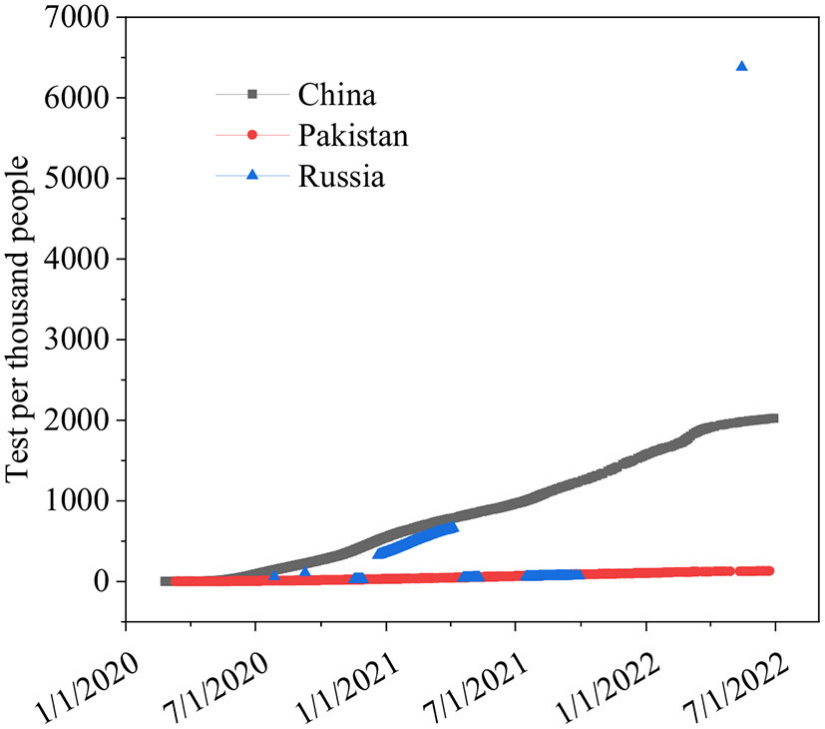
Daily COVID-19 tests per 1,000 people. Data source: COVID-19 testing. Comparisons between countries are compromised for several reasons. https://ourworldindata.org/coronavirus-testing#source-information-country-by-country, January 7, 2020–June 23, 2022.

**Figure 4:**
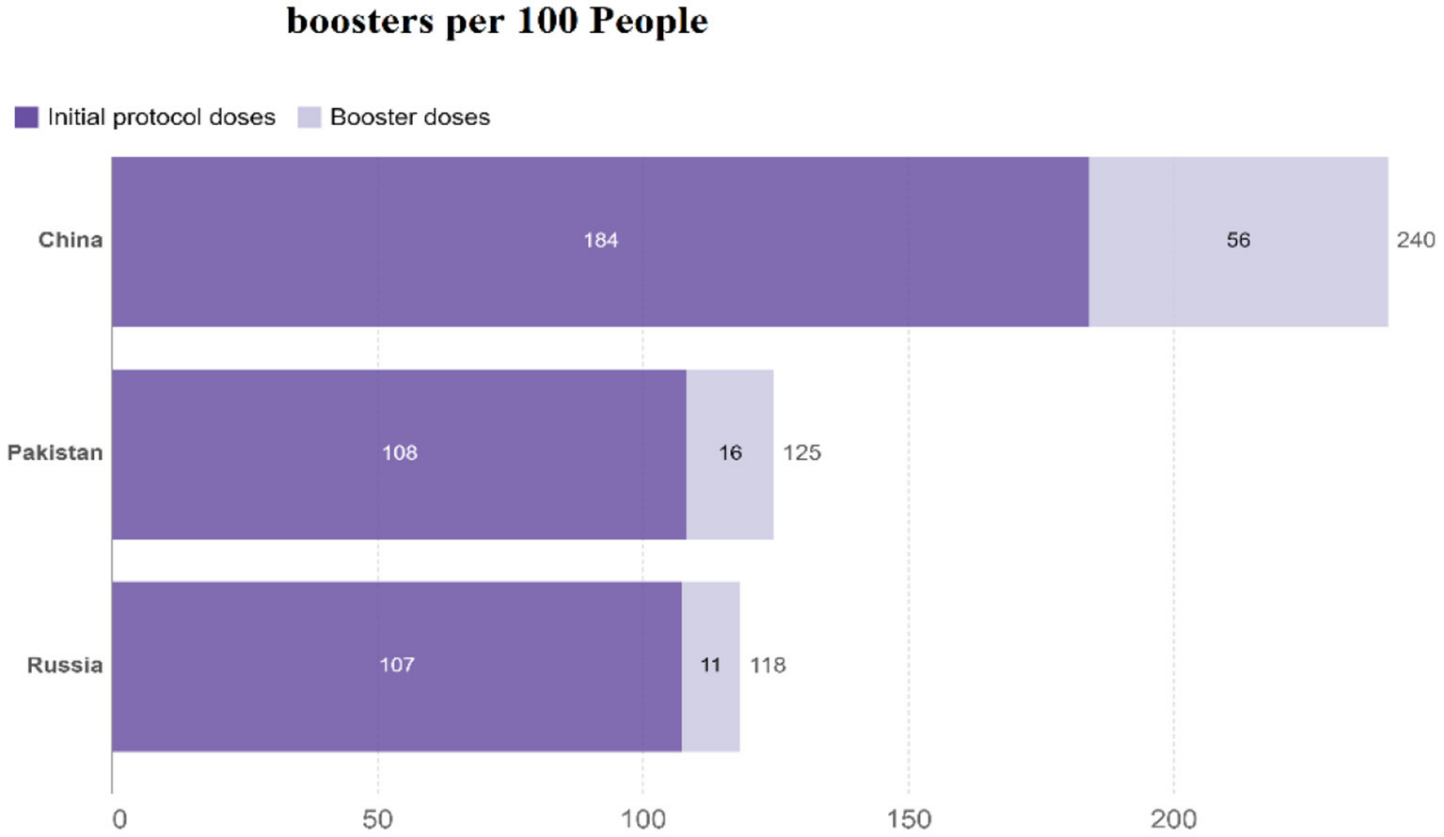
COVID-19 vaccine initial doses and boosters per 100 people. Data source: https://github.com/owid/covid-19-data/tree/master/public/data/vaccinations/locations.csv, only rely on figures that are verifiable based on public official, August 13, 2022.

**Figure 5:**
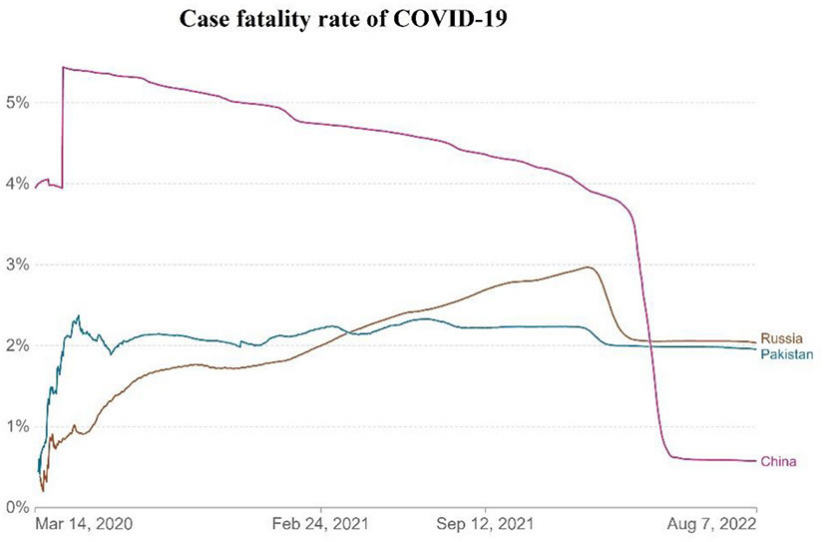
The daily case fatality rate of COVID-19. Data source: https://github.com/CSSEGISandData/COVID-19, raw data on confirmed cases and deaths for all countries is sourced from the COVID-19 data repository by the Center for Systems Science and Engineering (CSSE) at Johns Hopkins University. COVID-19 data repository by the Center for Systems Science and Engineering (CSSE) at Johns Hopkins University, March 14, 2020–August 14, 2022.

**Figure 6:**
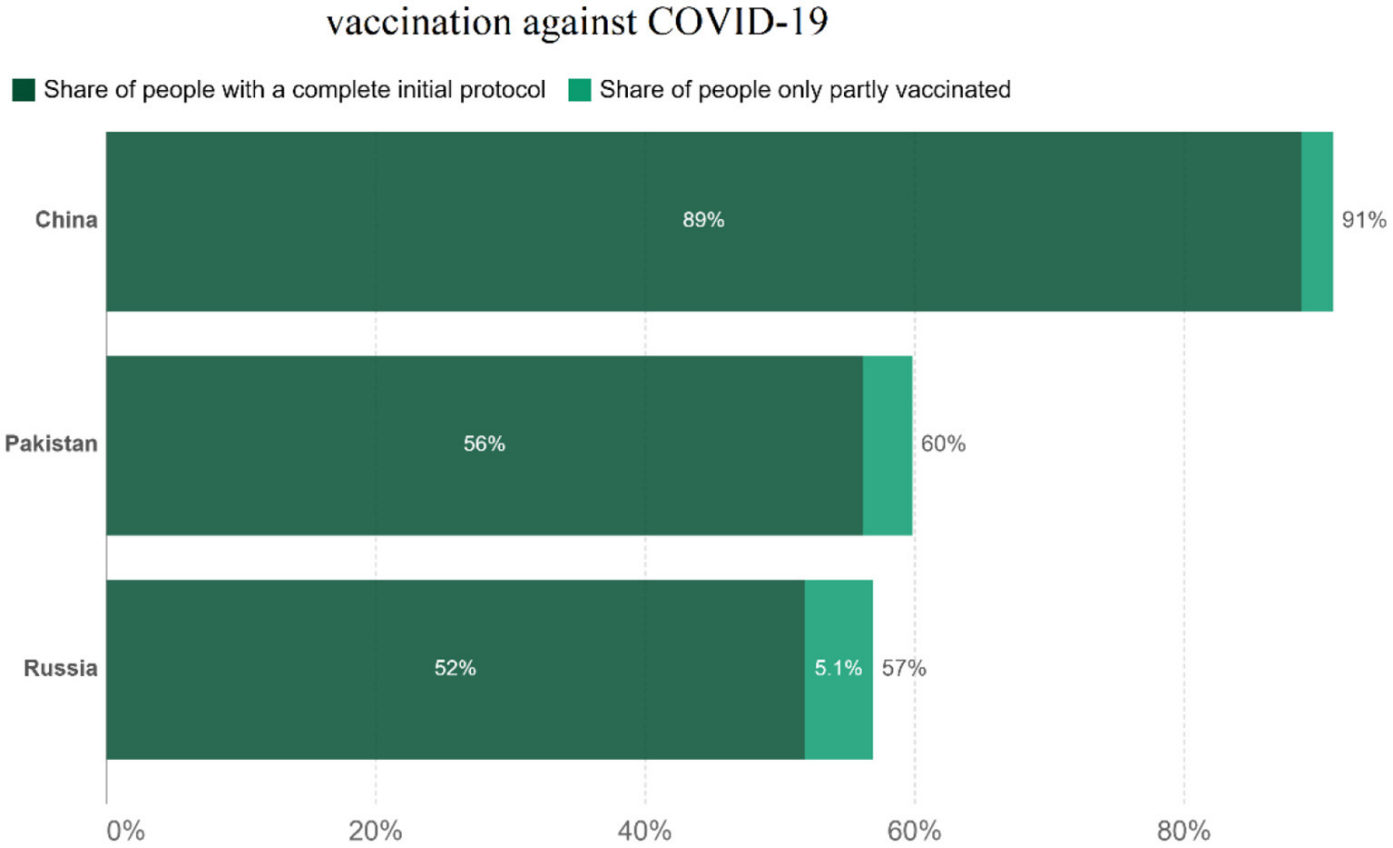
Share of people's vaccination against COVID-19. Data source: official data collated by Our World in Data, noted: alternative definitions of full vaccination, e.g., having been infected with SARS-CoV-2 and having one dose of a protocol, are ignored to maximize comparability between countries 14, 2022.

**Figure 7:**
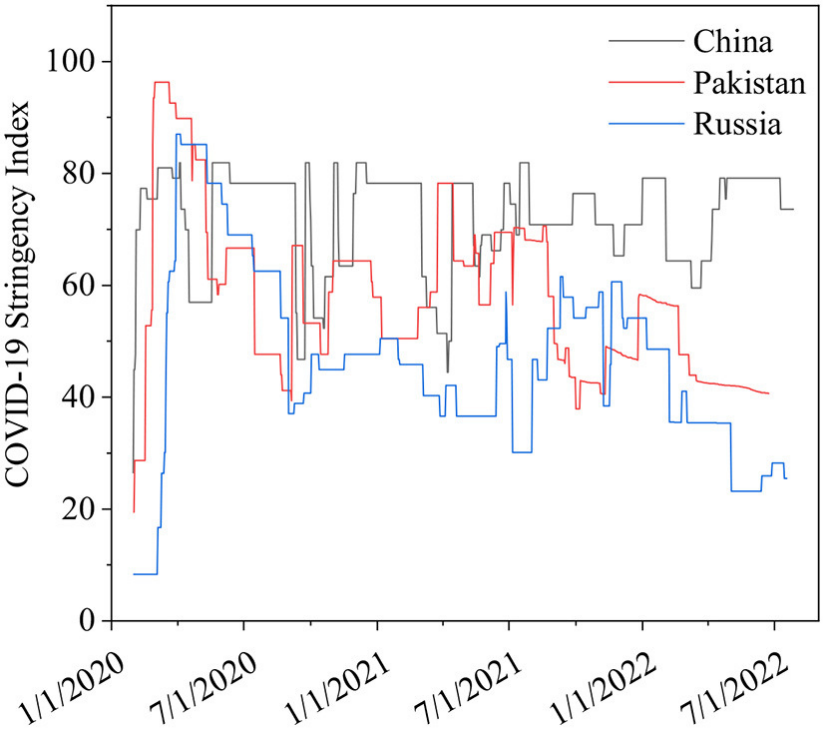
Non-Vaccination and Vaccinated Indices. Data source: Hale et al. (72), February 3, 2020–August 14, 2022.

The differences in China's and other nations' policy reactions to COVID-19 have sparked a lot of debate. While most other governments preferred enforced large-scale social separation lockdowns, China relied primarily on voluntary suggestions (70). Comparing China's COVID-19 mortality to Pakistan's and Russia's is instructive in terms of death statistics (67) (Figure 1), while all three countries' COVID-19 deaths per million people achieved epidemic suppression (a fall in the time-varying reproduction number Rt to below), mortality varied greatly, with daily new confirmed COVID-19 cases per million people data. China, Pakistan, and Russia initially exhibited similar epidemic trends, implying equal levels of virus seeding in each country (67) (Figure 2). By 2022, China's mortality rate will have increased 5-fold above Pakistan's, following the implementation of regulations between 2020 and 2022. Those Scandinavian nations had a lower fatality rate at the start of the epidemic than Russia, which suggests an earlier or stronger seeding of the virus. Prior to the implementation of regulations in India, the UK's cumulative mortality was comparable to that of China by 2022. Every day, a thousand people are tested for COVID-19 on a daily basis (Figure 3), the total number of doses administered, broken down by protocol or booster doses, is divided by China, Pakistan, and Russia's total population (Figure 4). We can see that China, Russia, and Pakistan all had a decrease in population contact rates at a faster pace than the other two countries, and showed a daily case fatality rate of COVID-19 on population mobility data, as well as the tracking of governmental initiatives and the COVID-19 case fatality rate (71) (Figure 5), there were fewer major changes in China's conduct and policies throughout this vital time of exponential expansion than there were elsewhere. However, the percentage of individuals vaccinated against COVID-19 in 2022 and the percentage of people vaccinated against COVID-19 stringency index statistics show that the vaccination rate has increased (Figure 6), the COVID-19 stringency index The Non-Vaccination and Vaccinated Indices explain the data in Figure 7.

The COVID-19 infection caused the deaths of over 380,000 people in Russia, 300,000 people in Pakistan, and 22,000 people in China, according to the WHO (73). China's fewer deaths than other countries during COVID-19 is likely due to the country's rapid adoption of digital technology. Large-scale lockdowns and curfews by the Chinese government have caused hospitals, clinics, and medical centers to close, even at the local level (74). This has stopped production lines, put education on hold, and kept millions of suspected patients from seeing doctors (75). Social separation has become the new social norm, with all offline activities suspended and personal relationships reduced (75). Because of the huge growth of online activities in the past few years, people can now work from home, learn from home, and even get medical care without ever having to leave their homes (75). As a side effect of the pandemic catastrophe, individuals may now communicate in cyberspace without touching one another (75). This is to keep the economy, social, educational, and medical communication moving in order to avoid illness and to keep people from falling sick (76). Compared to Pakistan, China's improved health protection laws and regulations are superior. Both Russia and China have a lot to learn from each other. Public health rules and regulations must be strengthened and improved immediately in both nations. Pakistan and Russia need to make changes to their public health laws and their laws and support systems for preventing and controlling major pandemics. Recently, COVID-19 positivity in Pakistan now stands at 3.53% following the latest cases. The coronavirus infections spiked sharply after a period of relative calm, with Pakistan seeing a 1,000% surge in cases the day before (77). According to its figures, 779 people in the nation have tested positive for COVID-19 in the preceding (77). The Russian Federation has had a record-breaking 7.83 percent increase in the number of new coronavirus infections in the last few days, making it the country with the highest number of illnesses since the outbreak began (78). According to our World data, China's mainland had < 0.3 confirmed cases of COVID-19 that were spread locally (78).

Due to an increase daily in COVID-19 cases caused by highly infectious Omicron, Delta, Alpha, and more the Coronavirus subvariants in Russia and Pakistan, there is concern that the disease will spread across the globe as immunity decreases and the summer travel season begins (77). However, China is in full control of the rising number of COVID-19 cases. Both Russia's and Pakistan's governments should learn from China's experience in dealing with the world's most populous country in the fight against pandemic flu (79).

This graph depicts the daily testing frequency per thousand people. If a day's test count fluctuates, we provide it as a rolling average for the last 3 or 4 days (80). China conducts a wide range of tests. COVID-19 More nucleic acids are produced every day than are produced in Russia and Pakistan put together (81).

The total number of doses administered is broken down by whether they are part of the protocol or booster doses divided by the total population of China, Pakistan, and Russia (82).

The case fatality rate (CFR) is the ratio between confirmed deaths and confirmed cases; the CFR is a poor measure of the mortality risk of the disease; we explain in detail the case fatality rate on March 2, 2020, the first Russian case of COVID-19 was verified in Moscow (83). The number of COVID-19 cases began to rise rapidly around mid-April. As of August 7, 2022, Russia was ranked ninth in the world in terms of the number of COVID-19 instances, behind the United Kingdom and Italy (84, 85). COVID-19 cases totaled 18,485,632 in Russia, with 381,835 deaths, despite an ongoing battle against the disease's rapid spread (78). Pakistan has recorded 1,545,647 cases and a death toll of 30,438 (77). This is lower than Russia, but China has developed strategies that are more focused on reducing and eradicating COVID-19. As of this writing, there have been 227,272 confirmed cases of COVID-19 and 5,226 confirmed fatalities, fewer than Pakistan in China until 2022 (Table 1). It has been demonstrated that China's stringent control measures considerably reduce the spread of the epidemic, and Russia and Pakistan can benefit from China's experience in COVID-19 (86).

Vaccination programs for COVID-19 in “high-income countries” (HICs) have promoted their capability to secure contracts for favored supplies for several vaccines (87). Vaccine access would be much less certain for the rest of the world despite the G20's vow to ensure that vaccines against COVID-19 are distributed fairly around the world. There was a cumulative lookup for HICs to contribute a proportion of their vaccine doses (88). During the H1N1 influenza pandemic, there was a coordinated effort among HICs to make a vaccine to protect the world's poorest countries, including a pledge by China's foreign minister to donate a lot of China's vaccine supply; this was supported by the general public (89).

The stringency index is a composite measure based on nine response indicators, including school closure and travel bans, rescaled to a value from 0 to 100 (100 = strictest) (72). A simple average of each sub-index makes up the index for each jurisdiction in which our non-vaccinated and vaccine-protected indices exist. This is explained in the following, where *k* is the number of components in an index and *I*_j_ is the sub-index score for an individual indication. Component indicators with differentiated policies will be recorded using one of the following.

- In the NV or V, for those who are non-vaccinated or else those who have been vaccinated index, a different set of rules apply.
- There is no differentiation between the E (everyone) and N (non-vaccinated indexes) policies.

(1) $$\begin{array}{l} index=\frac{1}{k}\overset{k}{\underset{j=1}{\sum }}I_{j} \end{array}$$

This resulted in two versions of each index reporting the overall policy settings for the un-vaccinated and vaccinated people, respectively.


(2) $$\begin{array}{l} index=\left(index_{v}+index_{nv}\right)/2 \end{array}$$

Simple average, those who have received vaccinations and those who haven't are both included in the simple average index for a certain jurisdiction. The vaccinated have a score of v, while the unvaccinated have a score of NV, as shown. It's all laid out for here.

(a) Weighted Average

With a complete initial protocol helping of data from the Our World in Data vaccination dataset with gaps filled, this method uses the non-vaccinated and vaccinated population percentages to weight the index value.

- In addition to the cases and death variables, the CSV includes a field called “Population Vaccinated.”

For display purposes, fill in the gaps. The display logic for the population-weighted average is as follows:

- If no data is available before or equal to the date, - > 0% vaccination is assumed.
- If there is no fully vaccinated per hundred for a specific date, - > then pull forward the value from the last day it was present.

(3) $$\begin{array}{l} index=\left[\left(index_{v}*W_{v}\right)+\left(index_{nv}*W_{nv}\right)\right]/100 \end{array}$$

First, we calculated indices for those who were vaccinated and those who were not vaccinated in a specific jurisdiction. Finally, we divide these figures by the percentage of the population that has been vaccinated to the maximum extent possible. As shown below, the v index score for the vaccinated (i.e., those who have received the vaccine), the NV index score for those who have not received the vaccine, and the Wv and Wnv weights of the vaccinated and non-vaccinated populations, respectively, are all calculated (i.e., percent non-vaccinated). The denominator always equals 100, i.e., the sum of the percent of vaccinated and non-vaccinated people in a given country.

(b) Legacy Repo Indices

(4) $$\begin{array}{l} index=\frac{1}{k}\overset{k}{\underset{j=1}{\sum }}I_{j} \end{array}$$

A simple average of the different component indicators is used to calculate all of the indices in our heritage binder. If *k* is the number of components in an index, and *I*_j_ is the sub-index score for an individual indication, this is how it's explained below.

### 3.2. Describe of data source

Russian, Pakistani, and Chinese responses to the COVID-19 outbreak (a) for every million persons in the world, a new COVID-19 fatality is reported every day. Information on individuals gleaned from our massive database, (b) data collected by us every day from across the world on new instances of COVID-19 has shown the following (80). In (c) the number of persons, who Carry out hundreds of daily COVID-19 tests, (d) new cases of confirmed COVID-19 per million persons every day, (e) the case fatality rate (CFR) the ratio between confirmed deaths cases, (f) people vaccinated against COVID-19 2022, (g) COVID-19 stringency index and the can be a poor measure of the mortality risk of the disease (80).

Index of stringency in COVID-19 the stringency index is a scale from 0 to 100, with 100 being the harshest, based on a composite of nine reaction indicators, such as school closures, employment closures, and travel restrictions (80). At every given moment, the COVID-19 intervention stringency index is plotted on the right. There were stronger mobility and intervention stringency changes in Russia than in Pakistan or China, although they occurred a key few days later than in Pakistan.

Based on this qualitative assessment of mortality patterns, we will do a counterfactual analysis to determine the impact of intervention efficacy and timing on the ultimate death toll in each nation. We've decided to concentrate our efforts on three countries: Russia, Pakistan, and China. Because of their close proximity and similarity in demographics, social structures, and economic conditions, Pakistan and China have seen comparable early COVID-19 death rates. Policy responses to COVID-19, on the other hand, varied dramatically across the two nations, and COVID-19 mortality patterns began to diverge by the end of 2020. Russia is included in our research since it is a northern Eurasian nation that has had similar COVID-19 death rates to China while implementing control measures similar to those in Pakistan.

Consider the speculative possibility that China's COVID-19 management strategy had an impact on the outbreaks in Pakistan and Russia. If the two nations' actions had been reversed, how could the China pandemic have been impacted as a result? Our goal is to shed light on how the timing and efficacy of actions influence a country's illness burden in order to help future governments make better decisions. Counterfactual modeling is necessary to answer this question since randomized trials of population-wide strategies are not viable due to the unique nature of each country. There are no previous assumptions regarding the success of specific policies in our study, and we retain apparent disparities in the paths of epidemics before social distancing measures were implemented in each country.

For the time period between now and 2022, we evaluate the first and last waves of transmission, which include the implementation of measures to reduce transmission and the subsequent relaxation of those regulations. A semi-mechanistic transmission model and a Bayesian statistical strategy for propagating uncertainty are used to evaluate each counterfactual scenario. In spite of the many disparities between countries, our study provides useful guidance for future COVID-19 pandemic control efforts.

## 4. A discussion on comparison of law, policy, and governance for COVID-19 prevention and control of Russia, Pakistan, and China

The COVID-19 pandemic has quickly come to represent the biggest global infectious disease crisis and new legal reform in the legal system since the China, Pakistan, Russia, and other countries' influenza pandemic (90–92). Its high infection rates across borders, diversifying variants, and increasing contagiousness and lethality have made it a truly planetary disaster (90). It is evident, however, that the pandemic is considerably more than a natural or biological tragedy. It quickly spread around the world, took advantage of economic inequality and social insecurity, and brought to light the failures of planning for resilience, tourism, investment, and travel (93). All of these things show how important market mechanisms are in reducing and amplifying the damage caused by the pandemic.

### 4.1. Various initiatives by the federal and provincial governments of Pakistan

After the 18th amendment in the Constitution of Pakistan, 1973, health law and governance are the responsibility of provincial governments (94). Nevertheless, the federal government receives international aid and collaborations programs through which it supports the provincial governments (94). Given that, the federal government collaborated with global and regional agencies in the wake of COVID-19 and supported the provincial government through the formation of a cooperative framework under the auspices of the National Command and Operation Center (NCOC) (95). The provincial governments formed their own sets of legislation, namely Infectious Disease Prevention and Control Ordinances under the previous laws for prevention of epidemics known as Public Health (Emergency Provisions) Ordinance 1994 (96). Furthermore, the federal government of Pakistan, with the collaboration of international organizations, including WHO, took necessary precautions and different measures against the COVID-19 pandemic, which not only ensured containment of disease spread risk but also fulfilled its responsibility as a state toward its population's safety (97). The pandemic has also exposed the limitations and institutional weaknesses within the criminal justice system, underscoring the need for implementing timely and adequate response strategies to emerging issues (98). The police, being the first responders, are facing various challenges related to the protection of their staff, maintaining law and order, enforcing lockdown and cases of dismissal.

It is the mission of the United Nations Office on Drugs and Crime Pakistan (UNODC-Pak) to assist and strengthen the rule of law so that a criminal justice system is fair, secure, peaceful, and inclusive can be established, where institutions uphold the rule of law while also providing citizens with high-quality services that are timely and transparent (99). The COVID-19 pandemic has changed and disrupted the normal functioning of the Criminal Justice Institutions (CJI) in Pakistan (99). UNODC recognizes this and has rapidly developed strategies and plans to support the CJI to adapt to and better manage the crisis. A weekly strategic coordination platform has been set up along with criminal justice stakeholders to discuss challenging issues during the current crisis and embark on practical solutions to respond to the COVID-19 crisis. The unprecedented nature of the outbreak of Coronavirus Disease 2019 (COVID-19) has also opened up opportunities, including the one utilized by the UNODC-Pakistan to ingeniously reconfigure its strategy for supporting Pakistan's rule of law changes based on its engagement approach (100).

The government of Pakistan took different initiatives to control the COVID-19 spread. After declaring the COVID-19 epidemic, Pakistan International Airlines (PIA) decided to halt flight operations between Pakistan and China till January 30, 2020 (101). In response to China's rising COVID-19 positive rate, Pakistan's Civil Aviation Authority created screening stations at four major airports for all passengers traveling from China. In addition, on March 21, 2020, the Karachi airport introduced screening desks for all international and domestic visitors (102). Furthermore, inbound traveling through trains and local and private transport was initially put under strict scrutiny; the passengers were inspected for COVID-19 details and were recommended for self-isolation. The government also restricted attendance in public offices, schools, colleges and universities in the beginning as a measure to effectively control COVID-19. The mosques and other religious places were placed under strict guidelines, and the attendees were reduced significantly in order to contain the spread (99).

As part of Pakistan's Rs. 1.2 trillion economic relief package, the government of Pakistan pledged 150 billion rupees (852,412,200 dollars) to help low-income groups like laborers; 280 billion rupees ($1.76 billion) to help buy wheat; and 100 billion rupees (63 million dollars) to helped exporters (with loan interest deferred) (103). Customers of gas and electricity may now pay their bills in many installments according to a policy introduced by the state. It was agreed by the Government of Pakistan to expand the national income support program. Previously, the monthly stipend was Rs. 2,000, but now it's Rs. 3,000, the Ehsaas program fund of the government was divided among the 5.2 million recipients who had been enrolled (later, this number increased due to increased unemployment). Healthcare workers were offered a unique bundle of relief. A martyr's kit would be sent to the families of everyone who died while working on COVID-19. In a meeting on March 31, 2020, the government of Pakistan authorized 100-billion-rupee supplemental funding for COVID-19's Emergency Relief Fund. Special packages worth Rs. 12 million have been offered for the benefit of low-income households through the district administration's Ehsaas and Kafalat programs (103). With the one-time dispensation, the financial aid term lasted for 4 months. After biometric verification, Kefala's partner banks, HBL bank and Alfalah bank limited, provide the Rs. 12,000 to the recipients. According to the government, 1.77 million impoverished households received a total of Rs. 22.466 billion in aid (103).

### 4.2. Initiatives by the Chinese government to combat the epidemic

A cluster of pneumonia patients with an unclear etiology had been recorded before the massive outbreak in Wuhan, but no one paid notice. The government was slow to realize the seriousness of the situation. In the face of the epidemic, the Chinese people have shown tremendous strength and zeal (95). People responded favorably to the government's appeals for help and cooperation. Traffic control began with peasants in Henan Province, a northern neighbor of Hubei, digging large holes in the road as barriers to prevent people from getting to their destinations (81). It demonstrated the openness of the public to become involved in public affairs. The Spring Event is the most significant festival in Chinese society, and it was around this time that the lockdown and quarantine were first implemented that families were convinced to forego the traditional manner of celebrating and instead send wishes through text (74). The general public's way of life has shifted dramatically as a result of this. Even if just a few incidents are recorded, Chinese law mandates city-wide lockdowns, making this one of the most stringent regimes in the world.

The Mayor of Wuhan believed that government, according to the law, was still the underlying principle guiding the conduct of his government; however, the legislation or flaws therein limited their ability to respond appropriately to this catastrophe (59). As a result, many people believe that the law and the rule of law should play an important part in the battle against COVID-19 and urge that infectious disease control be done in accordance with the law and rely on forces under the rule of law. Part of China's general legal framework for emergencies, the Law on Prevention and Treatment of Infectious Diseases, was passed in 1989, but the legal framework for public health emergencies was not fully established until after the SARS epidemic in 2002/2003 and subsequent H1N1 and H7N9 avian flu pandemics in 2009 and 2013, respectively. Many other areas of Chinese law have a similar structure: primary national legislation, implementation regulations and measures issued by relevant ministries, judicial interpretations of the Supreme People's Court and Supreme People's Procuratorate, as well as local implementing rules (104).

(a) Laws adopted by the National People's Congress's Standing Committee

- The 1989 Infectious Diseases Prevention and Treatment Law (issued by the SCNPC in 1989, revised in 2004 and 2013, hereinafter referred to as the Law on PTID) (105).
- The Emergency Response Act of 2007, as amended (issued by the SCNPC in 2007, hereinafter the Law on ER) (106).

(b) The State Council's regulations

- Public Health Emergency Response was a regulation (2003, issued by the State Council in 2003, revised in 2011) (107).

(c) Measures delivered by the Ministry of Health

- Preventing and Treating Infectious Diseases: Measures to Put into Practice the Law (issued by the Ministry of Health in 1991, hereinafter Implementing Measures for the PTID Law) (108).
- Infectious disease and public health emergency data reporting and monitoring administrative measures (issued by the Ministry of Health in 2003, revised in 2006, hereinafter Measures on Information Reporting) (109).
- The Public Health Emergency and Infectious Disease Information Dissemination Program (issued by the Ministry of Health in 2003; revised and re-issued by the State Commission on Health and Population Planning in 2006) (110).

China's Regulation on Open Government Information (approved by the State Council in 2007 and updated in 2019) mandates that governments provide information that might potentially harm the public's health, among other things. Regulations on animal disease prevention and control, as well as quarantine laws at national borders, are also in effect (107). In addition, there was a substantial amount of regional legislation; there are several judgments and decrees issued by the local authorities in reaction to the coronavirus crisis issued by the local legislatures. Most local implementing rules offer very little substance in terms of responses to such an emergency, as the national legal framework has already covered most issues. It was only after the city of Wuhan was placed under lockdown in early February 2020 that the most current judgments and instructions were made in conformity with national rules and regulations (59).

The broad and ambiguous wording used in these judgments and directives makes it difficult to understand how national legislation should be implemented (59). While it is not uncommon for the language of Chinese legislation to be general and vague, it is unclear whether the relevant authorities did so with full knowledge of the potential illegality of their decisions and orders, as the scope of their rule-making powers, especially in relation to the imposition of any restrictions on personal liberty and freedom, This is clearly limited by several national laws, including the Law on Law-Making (2000, amended in 2015), the Administrative Penalty Law (1996, revised in 2009 and 2017), and the Law on Administrative Compulsory Measures (2002, revised in 2015), which expressly limit this (2011) (111). Finally, there are many “opinions” issued jointly by local courts, local procuratorates, and local bureaus of public security (police), and these “opinions” mostly relate to penalizing violations of restrictions imposed locally. As a result, despite the fact that local laws and rules may influence the execution of national laws and rules, the national legal framework is ultimately responsible for determining the reactions to and implementation of the coronavirus problem.

### 4.3. The government of Russia's initiatives to tackle the pandemic

Late in the COVID-19 pandemic reaction was Russia. At the end of December 2019, a few little steps were taken, but it wasn't until March 30, 2020, that the government announced a variety of quarantine-like limitations in response to an uptick in illnesses (112). A “non-working period” in which people were compelled to stay at home and the state would pay them was mandated in all situations, regardless of where they lived (113). Most of the regional authorities decided to reinstate a small number of limitations in November 2020 as a result of a fresh outbreak of diseases. Regional differences exist in these (114). Despite the purported benefits of a more centralized government, the pre-Putin era's remaining regional autonomy is being used more and more in the present crisis management. As a result, the President's responsibility for unpopular policies can be transferred to local governors (115). Most of the small new social support programs that were put in place in 2020 can be found at the federal center.

Throughout the COVID-19 epidemic, basic rights were guaranteed by regional rules. But because Russia's health care system has been in trouble for a long time because of budget cuts, there was a strong argument that the state's failure to give health care workers the right safety gear could have violated their right to live (116). Anti-virus laws have been used to suppress public protest, thereby restricting the right to assemble, and “administrative arrest” powers have been used to temporarily jail individuals (117). As a result of the epidemic, Russia changed its laws against “fake news,” which hurt people's rights to speak and write freely. Laws restricting registration and activities, as well as the executive's persistent intervention, limit democratic institutions' ability to function effectively (118). The country's weak party structure, which is heavily dominated by the “party of power,” United Russia, is another hindrance to real progress. The poor way democratic institutions work is made worse by a passive population and a civil society that can't deal with too much government control (112). Legislative provisions were often poorly enacted by inefficient management. This presents citizens with chances to take advantage of the state's weaknesses. During his time in office, President Dmitry Medvedev spoke out against “legal nihilism” (112). However, efforts to keep the government from messing with the law did not work.

(a) New Russian laws passed during COVID-19 went into effect on April 1, 2020.

- To toughen administrative and criminal liability for violations of sanitary and epidemiological rules (114).
- To give the Russian government more power to declare a state of emergency and a high-alert regime (118).
- This provision provides the Russian government with the right to establish special rules for the registration and circulation of medicines and medical devices to prevent the spread of dangerous diseases (116).
- Violation of sanitary and epidemiological rules; or failure to comply with anti-epidemic measures during emergencies when a dangerous disease threatens to spread or when quarantine has been introduced within a specific territory; or failure to comply with legal requests or instructions of an authorized body regarding anti-epidemic measures (e.g., a quarantine instruction issued to a person who has come back from overseas) (119).

(b) The following fines are imposed in response to certain violations.

- To up to RUB 40,000 (EUR 470) for individuals (119).
- To up to RUB 150,000 (EUR 1,750) for company officials (119).
- To up to RUB 150,000 (EUR 1,750) for individual entrepreneurs (119).
- To up to RUB 500,000 (EUR 5,830) for lawful entities. Instead of paying fines, entrepreneurs and legal entities may have their business activity stopped for up to 90 days (119).

(c) The possibility of punishment

Violations of sanitary and epidemiological regulations are now subject to criminal prosecution. If sanitary and epidemiological rules are broken in a way that makes a lot of people sick or puts a lot of people at risk of getting sick, criminal responsibility can lead to one of the following punishments (120).

- Up to RUB 700,000 (EUR 8,165) in fines or the equivalent in salary for up to 18 months (120).
- Ineligibility to hold certain positions for a period of up to 3 years (120).
- Limitation of liberty for up to 2 years (120).
- Compulsory labor for up to 2 years (120).
- Imprisonment for up to 2 years (120).

(d) If the violation resulted in a person's death due to negligence

- A fine up to RUB 2 million (EUR 23,330) or in the amount of salary for up to 3 years (120).
- Restriction of freedom for up to 4 years (120).
- Compulsory labor for up to 5 years (120).
- Custody for up to 5 years (120).

(e) If a violation caused the death of two or more people by negligence, the sanctions

- Required labor for up to 5 years (120).
- Custody for up to 7 years (120).

According to Russian legislation, the terms and length of such a postponement will be established by the Russian government in accordance with the law (120). Generally speaking, these new rules are in line with existing legislation's criteria for force majeure and a reduction in rents if conditions for operating the property have worsened (117). At the same time, they provide the renter with assurances about the present state of affairs with COVID-19 as a basis for requesting a delay or decrease in rent. Waiting for a government act to explain how to acquire a discount or a delay is the only way to get a more in-depth explanation.

### 4.4. Global governance through global health law

WHO encouraged the formation of acts or legislation, similar to a constitution which might grant broad powers to the administration or certain institutions to take emergency actions (118). Some states have chosen to enact other COVID-19 laws in response to the contagion's specific combination of conditions, which will serve as the foundation for a variety of emergency actions.

For example, the Pakistani Government Act of 2020 includes new legislation by the National Command and Operation Center (NCOC) against COVID-19 (37).

COVID-19-fighting tactics are getting increasingly effective at the moment. Despite the fact that the virus is still growing, preventative and control measures in several nations have reached a point where they can be managed on a regular basis. For example, China's normalized COVID-19 management corresponds to a “containment target,” which entails the total cessation of COVID-19 transmission locally (17). Until recently, the current COVID-19 outbreak has been a common scenario, with most nations managing to keep the number of cases low. However, due to viral mutations, small outbreaks continue to occur in some nations and regions. Local epidemics are more likely to develop in large-scale public facilities, such as transportation and entertainment venues, which are crucial for everyday living. The many States decided to prohibit mass gatherings, close big shops and schools, and decrease the number of flights and cruises to prevent the spread of the virus in the early phases of the outbreak (17). These tight preventative and control methods have become a problem for public health as schools, companies, and other similar establishments steadily open up. It is important to figure out whether or not public facilities can be fully opened as normal and whether or not transmission can be managed in a timely and efficient way if there is an epidemic (108).

Despite a sustained rise in case numbers, most European countries have begun to relax coronavirus restrictions due to high vaccination rates and the development of omicron as a dominant variation with mild symptoms (65). Denmark has taken the lead in Europe in resuming normalcy, removing all virus restrictions as of February 1 and proclaiming the coronavirus “no longer a major threat to society” (34). As of February 9, the Swedish Public Health Agency eliminated all COVID-19 limitations and discontinued COVID-19 testing, while Norway is due to withdraw its COVID-19 measures on February 17 (34). On February 14 and March 1, there would be no more limitations on the coronavirus in Finland. The Prime Minister, Sanna Marin, has also stated that the country's boundaries will be relaxed at that time (121).

The aforementioned voluntary system could play a critical role in making compulsory licenses feasible for a COVID-19 vaccine. It requires stronger enforcement through global health law to ensure widespread participation of public and private actors and alignment of international trade law with global health imperatives. In this diverse national regulatory landscape, global health law could aid in the efficient and effective regulation of new vaccines; for a COVID-19 vaccine, the WHO Pre-Qualification Process (PQP) might function in place of national rules and expedite regulatory approval if the PQP is recognized by states (118). Developing new forms of global governance through the law was necessary to implement human rights and gain access to a COVID-19 vaccination. The reform of global health law to ensure universal access to a COVID-19 vaccine was required: facilitating funding and benefit sharing, easing intellectual property protections, and harmonizing national vaccine regulations.

COVID-19 vaccination can only be made available to the entire world if global health legislation reforms are implemented. We begin by outlining the critical role played by global health law in the provision of life-saving vaccinations. The piece then examines the human rights basis of global health law, focusing on universal access to immunization. We found that it was important to make legal commitments to make sure people could get vaccines before there was a scientific breakthrough. We did this by looking at the legal barriers that make it hard for people around the world to get vaccines and the global health law reforms that were needed to help people work together.

## 5. Conclusion

Some shortcomings in global health policy have already been identified by research and are being addressed by regional and national systems. The so-called “freedom” of the West puts pressure on its own healthcare system, which occasionally fails to provide care for its own population. On the other hand, developing countries like Russia, China, and Pakistan have complied with global health governance standards, which has aided them in creating strategies to combat pandemics around the world. Regionally and internationally, praise is expressed for how these nations have responded to COVID-19 through their laws and policies. The 19 COVID-19 control measures' global behavior and standards have altered as a result of the legislation produced in these nations. There are many things that may be learned from these nations, such as how to create laws, policies, and particular joint departments for the prevention and control of such diseases. According to the WHO, these nations also promoted their own policies in light of their achievements, which developed nations might choose to support in order to assist developing nations in establishing long-term health governance structures.

## Data availability statement

The raw data supporting the conclusions of this article will be made available by the authors, without undue reservation.

## Author contributions

All authors listed have made a substantial, direct, and intellectual contribution to the work and approved it for publication.
